# The pericentromeric heterochromatin of homologous chromosomes remains associated after centromere pairing dissolves in mouse spermatocyte meiosis

**DOI:** 10.1007/s00412-019-00708-6

**Published:** 2019-06-04

**Authors:** Craig Eyster, Hoa H. Chuong, Chih-Ying Lee, Roberto J. Pezza, Dean Dawson

**Affiliations:** 1 Oklahoma Medical Research Foundation, Oklahoma City, OK USA; 2 Department of Cell Biology, University of Oklahoma Health Science Center, Oklahoma City, OK USA

**Keywords:** Meiosis, Centromere-pairing, Chromocenter, Heterochromatin, SYCP1

## Abstract

In meiosis, crossovers between homologous chromosomes link them together. This enables them to attach to microtubules of the meiotic spindle as a unit, such that the homologs will be pulled away from one another at anaphase I. Homologous pairs can sometimes fail to become linked by crossovers. In some organisms, these non-exchange partners are still able to segregate properly. In several organisms, associations between the centromeres of non-exchange partners occur in meiotic prophase. These associations have been proposed to promote segregation in meiosis I. But it is unclear how centromere pairing could promote subsequent proper segregation. Here we report that meiotic centromere pairing of chromosomes in mouse spermatocytes allows the formation of an association between chromosome pairs. We find that heterochromatin regions of homologous centromeres remain associated even after centromere-pairing dissolves. Our results suggest the model that, in mouse spermatocytes, heterochromatin maintains the association of homologous centromeres in the absence crossing-over.

**Electronic supplementary material:**

The online version of this article (10.1007/s00412-019-00708-6) contains supplementary material, which is available to authorized users.

## Introduction

Faithful homologous chromosome segregation at the first meiotic division depends upon connections that tether homologous chromosome pairs. The connections are normally created by crossovers between the homologous partners (Bascom-Slack et al. 1997). Chiasmata, the cytological manifestation of crossovers, keep the partners connected as they become stably oriented on the metaphase spindle. Stable attachments are formed when opposing microtubules pull the partner chromosomes towards opposite poles of the spindle creating tension that stabilizes the kinetochore-microtubule attachments (Nicklas and Koch 1969; Nicklas 1997). This tension is transmitted across the connection created by the chiasma nearest to the centromeres. Consequently, mutations that eliminate recombination are invariably associated with increased errors during meiotic chromosome segregation (Klapholz et al. 1985; Dernburg et al. 1998; Klein et al. 1999; Baudat et al. 2000; Romanienko and Camerini-Otero 2000), reviewed in Sansam and Pezza (2015). However, individual chromosome pairs that have failed to become joined by crossovers can nonetheless segregate properly in some organisms. In *Drosophila* and yeast, a high proportion of non-exchange chromosomes (those without crossovers) partition correctly in meiosis I (Grell 1962; Dawson et al. 1986; Hawley et al. 1992; Davis and Smith 2003). Thus, these organisms have mechanisms, beyond crossing-over, that can promote proper meiotic disjunction. There are suggestions that this may also be the case in mammals. In mice, the majority of chromosomes in oocytes from a recombination-deficient mutant appeared to be spatially balanced on the spindle, as if there are mechanisms to partition equal numbers of chromosomes to each pole (albeit not the correct chromosomes) (Woods et al. 1999). In humans, while smaller chromosomes (21 and 22) fail to experience crossovers in about 5% of meioses (Oliver et al. 2008; Fledel-Alon et al. 2009; Cheng et al. 2009), they are estimated to non-disjoin in only < 1% of meioses (Tease et al. 2002; Oliver et al. 2008; Fledel-Alon et al. 2009). Therefore, it may be that non-disjunction in mammals, as in yeast and *Drosophila*, may reflect the failure of multiple mechanisms: first, failure to generate a crossover, and second, failure of one or more backup systems that promote proper segregation of achiasmate (non-exchange) partners (Oliver et al. 2008; Fledel-Alon et al. 2009; Cheng et al. 2009).

Mechanisms that partition non-exchange chromosome partners have been described in yeast and *Drosophila*. In these organisms, the centromeres of non-exchange chromosomes pair or cluster in meiotic prophase (Ding et al. 2004; Gladstone et al. 2009; Newnham et al. 2010; Takeo et al. 2011). In budding yeast, centromere pairing in meiotic prophase predisposes the non-exchange partners to segregate properly in anaphase and may also contribute significantly to the segregation fidelity of recombined chromosomes (Kemp et al. 2004; Gladstone et al. 2009; Newnham et al. 2010).

The manner by which prophase centromere pairing in these organisms promotes disjunction is unclear. Homologous partners become tightly aligned in meiotic prophase by a structure called the synaptonemal complex (SC) that runs along their aligned axes. In budding yeast, the SC disassembles from the chromosome arms in late prophase except at the centromeres where it mediates their pairing (Gladstone et al. 2009; Newnham et al. 2010). But this centromeric SC largely disappears before metaphase when chromosomes become attached to the microtubules (Gladstone et al. 2009), so the pairing it provides cannot be the basis for mediating bi-orientation of the centromeres on the spindle. In *Drosophila*, segregation of non-exchange partners also appears to depend on pairing of their centromeric regions in prophase (Karpen et al. 1996; Dernburg et al. 1996). Observations of non-exchange chromosome partners in metaphase I in *Drosophila* oocytes show that the centromeres are not directly paired during the bi-orientation process but instead may be connected by threads of pericentromeric heterochromatin (Hughes et al. 2009). Connections between the pericentromeric regions might be formed when the centromeric heterochromatin of the homologous partners comes together in meiotic prophase (Giauque and Bickel 2016). Together, these results suggest the model that tight centromere pairing in prophase may allow the formation of heterochromatic chromatin connections that can then promote bi-orientation in metaphase.

In mouse spermatocytes, homologous partners experience a period of prophase centromere pairing (Bisig et al. 2012; Qiao et al. 2012). As in budding yeast, the pairing is mediated by SC components at the centromeres after SC disassembly. The centromere pairing holds the centromeres of both exchange and non-exchange partners in close proximity until it dissolves before prometaphase, with the removal of the SC components (Previato et al. 2018). Cytological evidence suggests the homologous centromeres may still be connected after centromere pairing dissociates, as thin strands or bridges of the chromosome axis component SYCP3 can sometimes be observed between the separated centromeres—suggesting the possibility of persisting connections between the centromeres even after SC components no longer keep the centromeres tightly together (Bisig et al. 2012; Qiao et al. 2012).

Here we explore the model that centromere pairing allows the formation of associations between the centromeric chromatin of the homologous partners. As in most eukaryotes, the centromeres of mouse chromosomes are flanked by blocks pericentromeric heterochromatin (Pardue and Gall 1970; Mouse Genome Sequencing Consortium et al. 2002; Martens et al. 2005). In early meiotic prophase, the pericentromeric regions of chromosomes associate in clusters called chromocenters (Jones 1970; Gall et al. 1971; Botchan et al. 1971), reviewed in Jost et al. (2012). Multiple centromeres cluster in each chromocenter (Berríos et al. 2010; Berríos et al. 2014; Hopkins et al. 2014), with homologous centromeres usually in different chromocenters (Takada et al. 2011).

Here we demonstrate that synaptonemal complex formation re-organizes pericentromeric associations, helping homologous centromeres move to the same chromocenters. After the SC-mediated centromere pairing dissolves in late prophase, the pericentromeric heterochromatin masses of the homologous partners remain associated, appearing to keep homologous centromeres linked, even for chromosomes apparently not tethered by chiasmata. Together, these observations suggest a mechanism by which centromere pairing in prophase might allow the formation of connections between homologous centromeres. Such connections might provide a link that helps non-exchange chromosomes become bi-oriented in the meiotic spindle.

## Results

### Pericentromeric heterochromatin moves from non-homologous to homologous associations through meiotic prophase

**Fig. 1:**
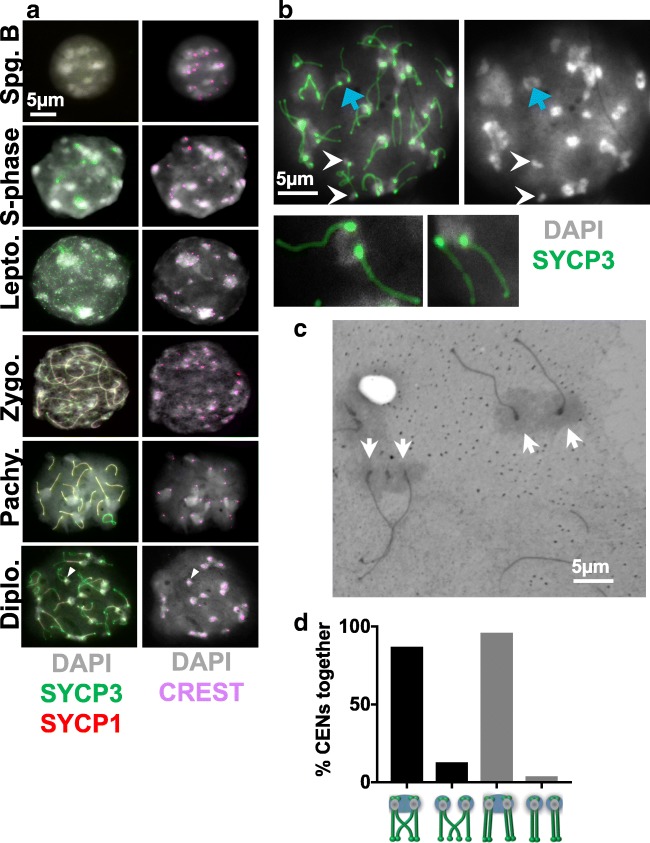
Dynamics of centromeric heterochromatin configuration during prophase in mouse spermatocytes. **a** Examples of wild-type spermatocytes at different stages of meiotic prophase I. Heterochromatin was visualized using DAPI. SYCP3 and SYCP1 immunostaining were used to stage spermatocytes and visualize the SC at paired centromeres. CREST served as a marker for centromeres. **b** Example of a late diplotene spermatocyte in which most centromere pairs share a common pericentromeric heterochromatin cloud. Blue arrow indicates an example of a centromere pair in a shared heterochromatin cloud. White arrowheads indicate a chromosome pair for which the pericentromeric heterochromatin is in separate clouds. Magnified chromosomes show details of pairs of homologs. **c** Electron microscopy of silver-stained wild-type diplotene spermatocytes showing examples of chiasmate (left) and apparently achiasmate (right) chromosomes connected by electron-dense pericentromeric heterochromatin. Arrows indicate centromeres of homologous pairs. **d** Quantitation of heterochromatin and centromere association in mouse spermatocytes. Individual chromosomes in chromosome spreads from diplotene mouse spermatocytes were scored for whether their centromeres (CREST) were in the same or different heterochromatin clouds. Chiasmate pairs *n* = 233, achiasmate pairs *n* = 26. Statistical comparison was with Fisher’s exact test. Scale bars = 5 μm except for magnified images of individual chromosomes

We monitored the behavior of pericentromeric heterochromatin in mouse spermatocytes to explore the possibility that interactions of the heterochromatin regions might promote proper meiotic chromosome segregation. All mouse chromosomes are sub-telocentric (the centromere is near one telomere), with a block of pericentromeric heterochromatin adjacent to the end that harbors the centromere (Pardue and Gall 1970; Mouse Genome Sequencing Consortium et al. 2002; Martens et al. 2005). These blocks of pericentromeric heterochromatin can be visualized cytologically as bright DAPI staining bodies, chromocenters, in the nucleus (reviewed in (Jost et al. 2012)). Mice have 20 pairs of chromosomes (19 pairs of somatic chromosomes and an XY pair in males); thus, complete pairing of homologous centromeres in pachytene spermatocytes would yield twenty-one centromeric signals (nineteen autosome pairs plus the X and Y), while completely dispersed centromeres would yield forty signals. We scored the number of heterochromatin signals (DAPI) in wild-type cells at advancing meiotic stages (S-phase through late prophase) (Fig. 1a). Centromeres were identified by their characteristic knob of SYCP3 staining that develops in late prophase (Moens and Spyropoulos 1995; Parra 2004) and by staining with CREST antibodies that recognize centromere proteins. As described previously (Berríos et al. 2010; Hopkins et al. 2014), from pre-meiotic stages through prophase, the centromeres cluster in chromocenters (Fig. 1a; Fig. S1). In early prophase (leptotene), there are fewer and larger chromocenters and they contain higher numbers of centromeres. By diplotene, the chromocenters have resolved to become smaller and significantly more numerous and harbor fewer centromere pairs (Fig. 1a; Fig. S1). In mid-diplotene, centromeres are usually tightly paired with their partners by a short remnant of persisting synaptonemal complex (Fig. 1a, diplotene, white arrowheads) (Bisig et al. 2012; Qiao et al. 2012). By late diplotene, this tight centromere paring dissolves, but the homologous centromere pairs remain within the same chromocenter, sometimes together with the centromeres of other homologous pairs (Fig. 1b).

This association of the pericentromeric heterochromatin of diplotene centromeres was confirmed by electron microscopy of silver-stained diplotene spermatocytes (Fig. 1c) which showed that the centromeres of homologous chromosomes remain in close proximity—connected by electron-dense clouds of pericentromeric heterochromatin.

### Centromeres of apparently achiasmate partners exhibit associations of pericentromeric heterochromatin

Previous work has shown that centromere pairing occurs as efficiently with achiasmate pairs as with chiasmate pairs (Previato et al. 2018). If the shared heterochromatin cloud that occurs when centromeres are paired can act as a tether to hold centromeres together, then centromere pairs should remain in the same heterochromatin cloud regardless of whether the homologous pair is tethered by a chiasma. To test this, we scored individual chiasmate and apparently achiasmate partners (those with parallel SYCP chromosome axes with no sign of axis exchange) in late diplotene chromosomes spreads. At this stage, centromere-pairing has dissolved (there is no more visible SC holding the centromeres together). We evaluated whether the homologous centromeres remained connected through a common heterochromatin cloud. We found there was no significant difference between the frequency of chiasmate or achiasmate centromere pairs remaining in a shared heterochromatin cloud (87% vs 96% respectively; *p* = 0.33) (Fig. 1d). This does not prove the centromeres are somehow tethered but is consistent with a model in which shared heterochromatin may be sufficient to keep partner centromeres joined, in the absence of chiasmata.

### Homologous pericentromeric associations are formed in late prophase

**Fig. 2:**
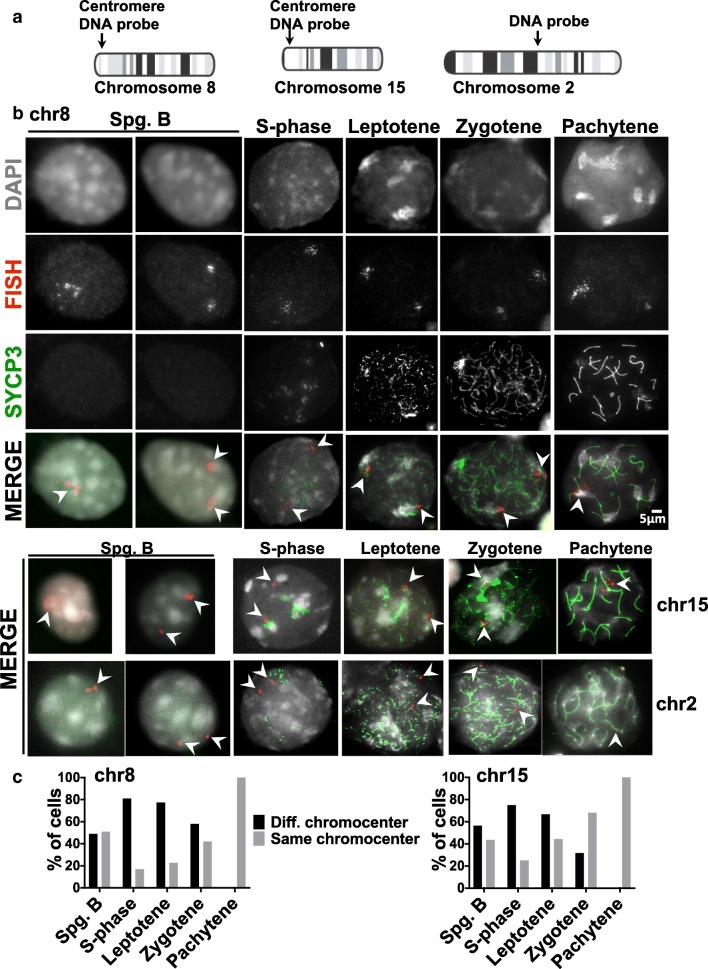
Homologous pericentromeric heterochromatin connections are established in zygotene and pachytene. **a** Chromosomal locations of FISH probes. The probes from chromosomes 8 and 15 are mainly unique sequences that map close to the centromeres. **b** Examples of spermatocytes showing FISH signals (arrows) using a chromosome 8 centromere-adjacent probe (top panels). SYCP3 immunostaining and DAPI signals were used for spermatocyte staging; DAPI staining was used to identify chromocenters. Bottom panels show merged images of FISH experiments using probes from the chromosome 15 centromere-adjacent region or the internal chromosome 2 region. **c** FISH images were scored for whether the chromosome 8 and 15 centromere-adjacent FISH signals were associated with the same or different chromocenters in chromosome spreads from different stages of meiosis I. Cells scored per meiotic stage for chromosomes 8 and 15 respectively were as follows: spermatogonia B 350, 287; S-phase 80, 59; leptotene 35, 30; zygotene 60, 66; pachytene 190, 127

Previous studies have suggested that in early prophase centromeres are in heterologous clusters that appear as large chromocenters and that by zygotene they have organized as homologous pairs (Takada et al. 2011). To directly track this progression, we used fluorescent in situ hybridization (FISH) to monitor the behavior of centromere proximal regions from chromosomes 8 and 15, and a control internal locus on chromosome 2, in cells at different stages of meiotic prophase (Fig. 2). Because mouse centromeres are composed of repeated major and minor satellite sequences, we used as FISH probes, unique sequences from chromosomal positions adjacent to the centromeres (in bacterial artificial chromosome vectors) (Fig. 2a). The probes correspond to approximately 200 kb of genomic sequence, so the signals typically appear as large, sometimes hazy, points. The FISH signals from the chromosome 8 and 15 centromere-adjacent probes were localized in, or adjacent to, the chromocenters while the internal chromosome 2 probe signals were not usually associated with the chromocenters (Fig. 2b). For the centromere probes, we scored the frequency with which they localized to the same or different chromocenters. In early prophase (leptotene), the homologous centromere-proximal FISH foci nearly always localized to different chromocenters, but by pachytene, they co-localized to a single chromocenter and were usually observed as a single diffuse spot around the synapsed centromeres (Fig. 2b, c). Thus, the early clustering of centromeres in chromocenters (leptotene) is not based on homology. This early clustering of pericentric heterochromatic regions is reminiscent of the homology-independent “centromere coupling” phenomena that occurs in early meiotic prophase in several organisms (Tsubouchi 2005), reviewed in Obeso et al. (2014).

### Homologous centromeres with more constitutive pericentromeric heterochromatin are more closely associated

**Fig. 3:**
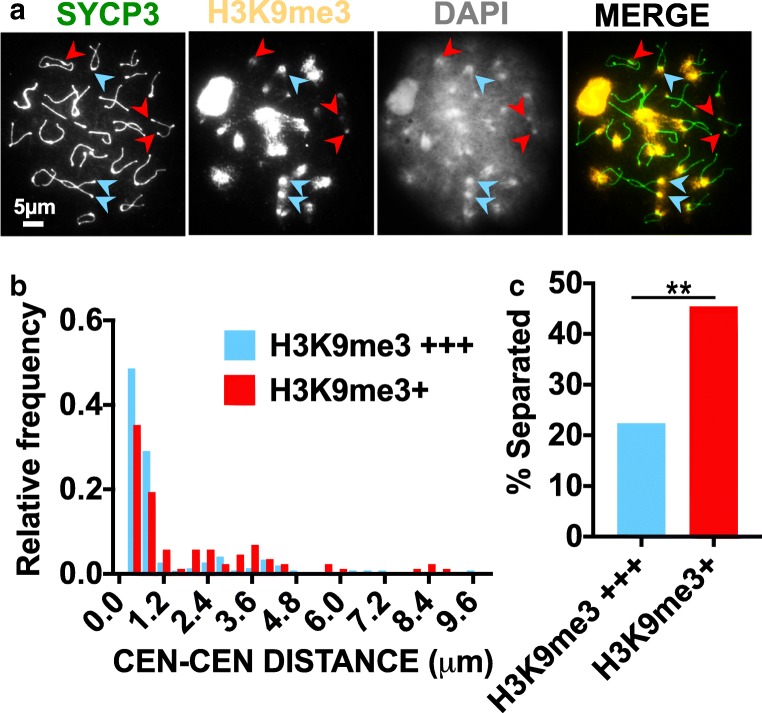
Centromeres with more heterochromatin are more likely to remain together. **a** Chromosome spreads were stained with antibodies against H3K9me3 to mark pericentromeric heterochromatin and SYCP3 to identify chromosome axes. Chromosomes were categorized as having bright or dim H3K9me3 staining, and the distances between the SYCP3 centromere knobs were measured. Blue and red arrowheads indicate examples bright and dim H3K9me3 staining. **b** Distances between centromere pairs (in 0.4 μm bins). *n* = 144 bright centromere pairs, 88 dim centromere pairs. **c** Centromeres farther apart than 0.8 μm were scored as separated. Centromere pairs with dim (+) H3K9me3 staining were significantly more likely to be separated than those with bright (+++) H3K9me3 staining (Fisher’s exact test, *p* = 0.0016)

If associations between blocks of pericentromeric heterochromatin help to keep homologous centromeres together, then those with more abundant heterochromatin might remain together more efficiently. To test this idea, we used the levels of methylated histone 3 lysine 9 (H3K9Me3) as an indicator of the amount of heterochromatin. H3K9Me3 is a post-translational modification of constitutive pericentromeric heterochromatin (Peters et al. 2001; Lehnertz et al. 2003) (Fig. 3a) and promotes the association of pericentromeric regions in *Drosophila* meiosis (Giauque and Bickel 2016). Chromosome spreads were stained with antibodies against chromosome axes and H3K9Me3, and the association of centromeres was scored as a function of the levels of heterochromatin in their chromocenter. As has been shown previously (Berríos et al. 2010; Hopkins et al. 2014), in late diplotene, homologous centromeres (identified by their SYCP3 knobs) although well separated are often in a cloud of shared H3K9me3-modified heterochromatin, and multiple homologous centromere pairs sometimes share a common heterochromatin cloud. There is considerable variation in the amount of heterochromatin at different centromeres by both DAPI and H3K9me3 staining (Fig. 3a). We categorized centromeres as having abundant or weak H3K9me3 staining then measured the distances between the centromeric SYCP3 knobs (Fig. 3a, b). Centromere pairs that were farther than 0.8 μm apart (the approximate maximal distance between aligned axes (Qiao et al. 2012)) were scored as “separated” (Fig. 3c). By this criterion, centromere pairs with low levels of heterochromatin were more likely to become separated in chromosome spreads (48% vs 27%; *p* < 0.01), consistent with the model that associations of the homologous pericentromeric regions keep the centromeres together.

### Homologous pericentromeric heterochromatin regions move to the same chromocenters late in synapsis

**Fig. 4:**
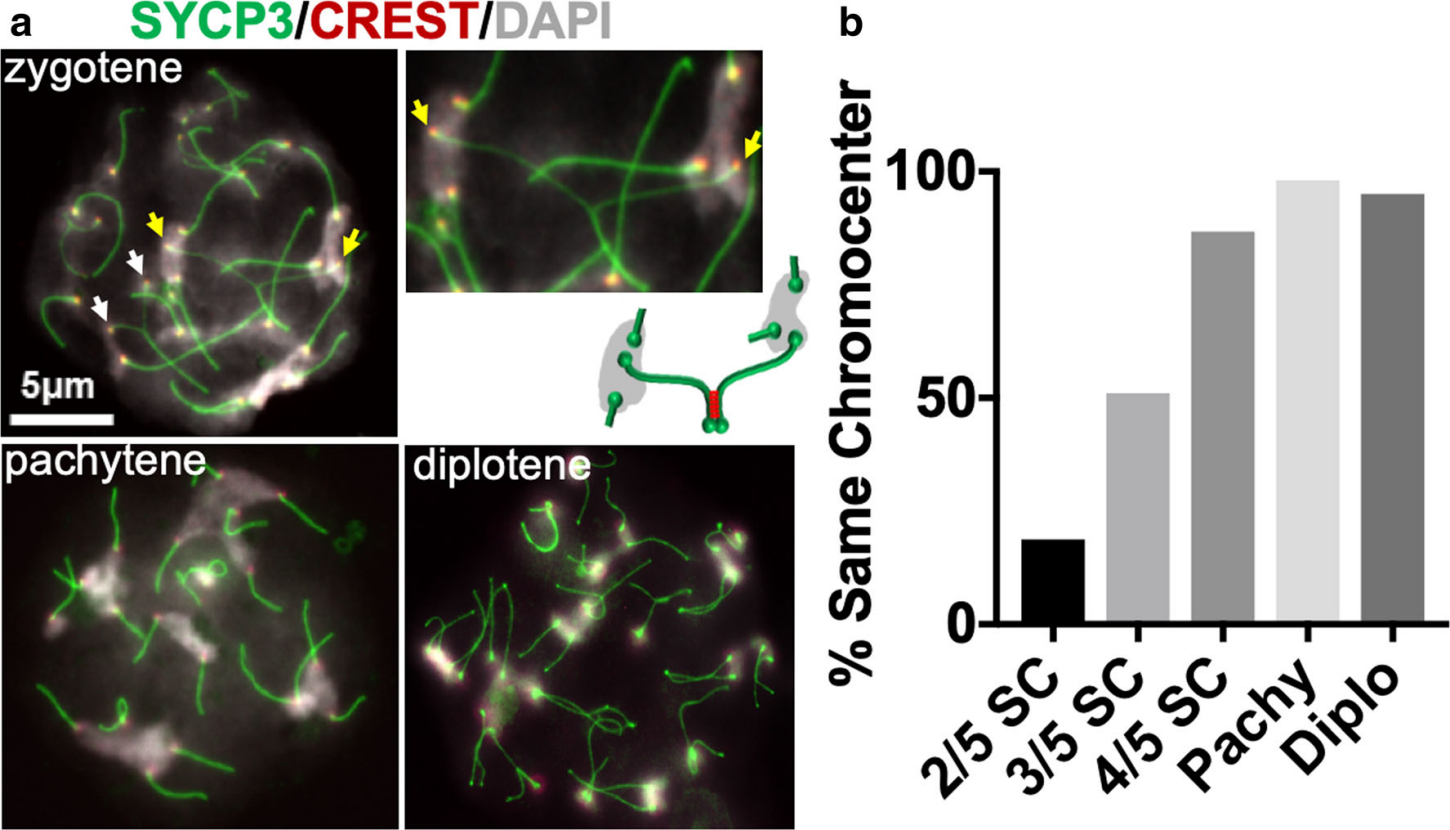
Pericentric associations move from non-homologous to homologous in meiotic prophase. **a** Representative chromosome spreads from spermatocytes in different stages of meiotic prophase. Staging was determined by the amount of synapsis. Spreads were stained to visualize chromosome axes (SYCP3; green), centromeres (CREST; red), and chromatin (DAPI; gray). In zygotene spreads, unsynapsed centromeres are frequently in different chromocenters. Yellow and white arrows indicate two examples of centromere pairs in different chromocenters. The magnified inset shows a centromere pair (yellow arrows) in different chromocenters. The cartoon illustrates the organization of this centromere pair. In the cartoon, green represents chromosome axes and red represents SC which is not stained in the chromosome spreads. In pachytene, homologous centromere pairs are usually in the same chromocenters but often with other centromere pairs. In diplotene spreads, chromocenters are often smaller with only one or two centromere pairs. Scale bar = 5 μm for all images except magnified images of individual chromosomes. **b** The individual chromosome pairs in zygotene, pachytene and diplotene spreads were scored for whether the homologous centromeres were in the same chromocenters. *n* = 100 chromosomes for each meiotic stage

The above results suggest the model that synapsis drives the re-organization of the pericentromeric heterochromatin into homologous clusters. Consistent with this model, previous studies of chromosome synapsis in mouse spermatocytes revealed that centromere regions are often the last to synapse (Bisig et al. 2012; Qiao et al. 2012). To determine when centromeres transfer from chromocenters shared with heterologous partners to ones shared with homologous partners, we evaluated whether partially synapsed homologous partners in zygotene cells (cells that are undergoing chromosome synapsis) have their centromeres in different or the same chromocenter (Fig. 4a, b). Individual chromosomes were scored for the ratio of the length of synaptonemal complex (SYCP1) versus the length of the chromosome axis (SYCP3). For chromosomes in the very early stages of synapsis (with SC extending only two-fifth the length of the axis), the homologous centromeres were rarely in the same chromocenter (Fig. 4b) and as the SC increased to full-length (in pachytene), the centromeres moved completely into shared chromocenters (Fig. 4a, b). Diplotene cells, in which SC was completely disassembled, continued to show homologous centromeres sharing chromocenters, though sometimes they shared the chromocenters with another homologous pair (Fig. 4a, b).

### SC assembly is necessary to establish homologous pericentromeric heterochromatin associations

**Fig. 5:**
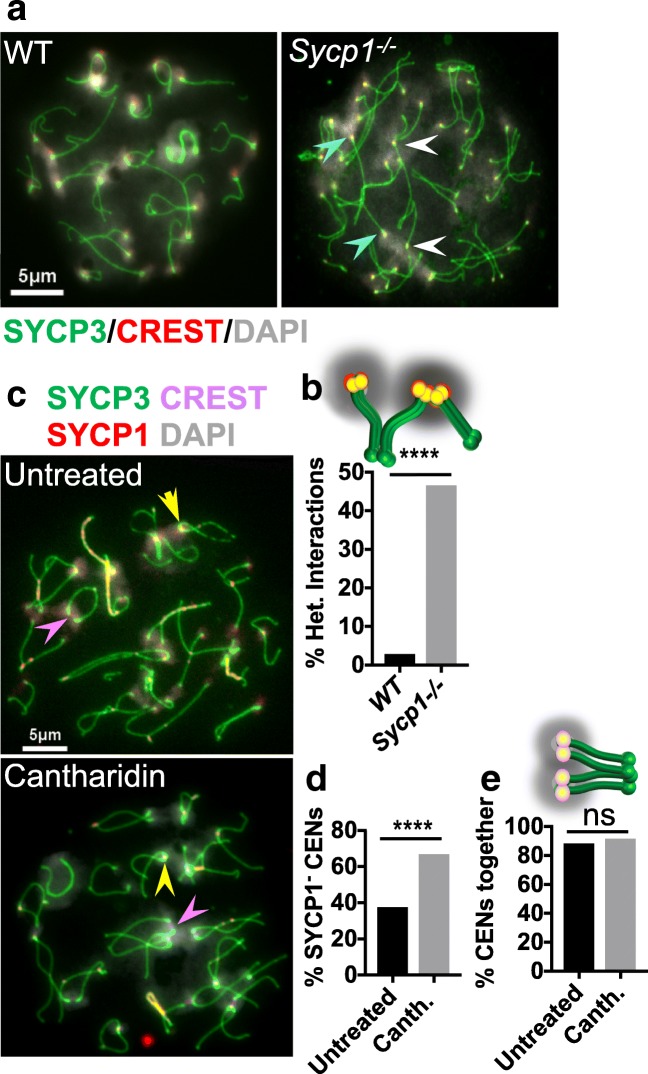
The SC is required for establishment but not maintenance of homologous heterochromatin-mediated centromere connections. **a** Chromosome spreads from diplotene wild-type and *Sycp1*^*−/−*^ spermatocytes were evaluated to evaluate the role of synapsis in the merging of homologous pericentromeric regions into a shared chromocenter. Cells were stained with DAPI (gray) and antibodies against SYCP3 (green) and CREST (red). Blue and white arrowheads indicate examples of two homologous centromere pairs that are in separate chromocenters. **b** Quantification of the frequency of chromosomes that had their centromeres in chromocenters with heterologous partners rather than in the same chromocenter. *n* = 500 chromosomes for wild-type and 290 for *Sycp1*^*−/−*^. *p* < 0.0001, Fisher’s exact test. **c** Examples of chromosome spreads from cultured diplotene spermatocytes, with or without a three-hour treatment with 30 μM cantharidin. Yellow arrowheads indicate examples of cells with persisting SYCP1 mediating centromere pairing. Pink arrowheads indicate examples of homologous centromere pairs with no detectable SYCP1. **d** Quantification of the percentage of chromosome pairs that were negative for SYCP1 (SYCP1^−^). *n* = 160 chromosomes from both untreated and cantharidin treated spermatocytes. *p* < 0.0001, Fisher’s exact test. **e** Quantification of the percent of homologous centromere pairs that were together (sharing the same chromocenter) in chromosome spreads from untreated or cantharidin treated spermatocytes. *n* = 160 centromere pairs for untreated (ten nuclei) and 160 centromere pairs for cantharidin treated (thirteen nuclei). *p* = 0.80, Fisher’s exact test. Scale bars = 5 μm

The above results suggest that SC assembly helps to pull centromeres out of different chromocenters that are shared with heterologous centromeres and into chromocenters shared with their homologous centromeres. This predicts that in mutants that are defective in synapsis, the centromeres will remain in chromocenters with heterologous partners late into prophase. To test this, we analyzed the dependence of the formation of homologous pericentromeric associations on SYCP1 (Fig. 5a). Chromosome spreads from wild-type and *Sycp1*^−/−^ spermatocytes that exhibited diplotene-like chromosome morphologies were scored for whether homologous centromere pairs were in the same or different chromocenters. In wild-type diplotene cells, the vast majority of centromeres were found to reside in chromocenters shared with their homologous partner (Fig. 5a, b); only 14 of 500 chromosomes scored (3%) had their homologous centromeres separated into different chromocenters. In the *Sycp1*^−/−^ spermatocytes, the homologous chromosome axes become aligned (Fig. 5a), as was described previously (de Vries et al., 2005). However, in sharp contrast to wild-type cells, we observed a high number of *Sycp1*^*−/−*^ spermatocytes in which the centromeres of the two homologs were in different chromocenters (135 of 290 (47%) chromosomes scored) (Fig.5a, b). We conclude that in *Sycp1*^−/−^ mutants, pericentromeric regions do not undergo the heterologous to homologous transition, even though the chromosome axes do become aligned. Although homologous pericentromeric regions are not efficiently placed into the same chromocenters in *Sycp1*^−/−^ mutants, the large chromocenters seen in leptotene cells are broken into smaller more numerous chromocenters (Fig. S1 C). Thus, complete synapsis is not required for the dissolution of large chromocenters that occurs as cell progress in meiotic prophase (Fig. S1).

### Pericentromeric associations persist after SC disassembly

Once the homologous pericentromeric regions become aligned through synapsis, is SYCP1 still required for the persistence of the pericentromeric associations? To test this, we took advantage of previous studies showing that inhibition of PP2A phosphatase drives SC disassembly in cultured spermatocytes (Wiltshire et al. 1995). SC disassembly is driven in part by phosphorylation (Tarsounas et al. 1999; Sourirajan and Lichten 2008; Sun and Handel 2008; Jordan et al. 2012; Argunhan et al. 2017), reviewed in Gao and Colaiácovo (2018), and PP2A presumably acts in prophase to reverse critical phosphorylations that drive SC disassembly. We treated cultured spermatocytes with the phosphatase inhibitor cantharidin (Honkanen 1993) and then examined chromosome spreads to determine first, if cantharidin promotes the loss of the persistent SC at paired centromeres in diplotene spermatocytes (Fig. 5c). In diplotene cells, cantharidin treatment significantly increased the numbers of centromere pairs without SYCP1 (Fig. 5d; SYCP1^−^ CENs). Thus, cantharidin treatment in diplotene causes the loss of centromeric SYCP1 after the centromeres have been paired. This allowed us to ask whether centromeres that are no longer directly tethered by SC continued to share a common chromocenter. The loss of SYCP1 did not result in a separation of the centromere pairs; instead, they remained joined by a shared cloud of heterochromatin (Fig. 5e). These results indicate that heterochromatin connections have been already established by mid-late diplotene, when SYCP1 remains at the centromere to mediate centromere pairing. The results also indicate that heterochromatin connections between homologous centromeres are stable in the absence of centromeric SC, raising the possibility that these connections might provide a link between homologous centromeres that could contribute to their bi-orientation as they transition from prophase into pro-metaphase.

## Discussion

### Role for heterochromatin in maintaining meiotic chromosome alignment

In *Drosophila* females, pericentromeric chromatin has been implicated in helping promote the segregation of homologous chromosomes, even if they fail to be joined by chiasmata (Karpen et al. 1996; Dernburg et al. 1996). In *Drosophila* females, chromosomes that fail to undergo recombination are connected by heterochromatic threads during prometaphase I as chromosomes orient on the meiotic spindle. These threads have been proposed to serve as a connection between the partners that may help them to bi-orient on the spindle (Hughes et al. 2009). Additional evidence for the conservation of heterochromatic threads connecting chromosomes during meiosis comes from *Drosophila* and crane fly sperm (LaFountain et al. 2002; Hartl et al. 2008). The results presented here demonstrate that heterochromatin also plays a role in promoting meiotic centromere interactions in the mouse and that these interactions are consistent with a role in promoting proper meiotic segregation, especially of achiasmate partners.

### Origin and regulation of heterochromatin-mediated centromere clustering early in prophase

**Fig. 6:**
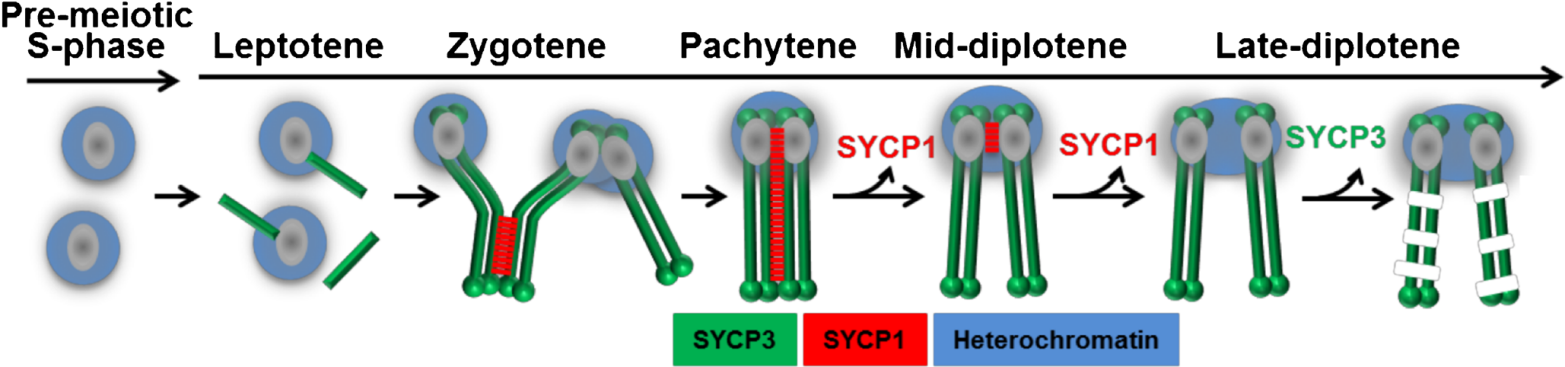
Cartoon summarizing the behavior of homologous pericentromeric regions during meiotic prophase

Observations in a wide range of organisms show that very early in the meiotic program (leptotene), before homologous pairing occurs, centromeres associate in pairs or clusters independent of sequence homology (reviewed in (Obeso et al. 2014). This is termed centromere coupling (Tsubouchi 2005). This work confirms previous observations of centromere clustering in the mouse suggesting that it resembles centromere coupling (Berríos et al. 2010). Our results show that as cells progress through prophase, centromeres move from large chromocenters bearing multiple heterologous centromeres to smaller chromocenters that include their homologous partners (Fig. 6). The mechanism by which the pericentric heterochromatic regions become re-organized has not been clear, but our results suggest that it is driven by synapsis. First, the homologous centromeres move to shared chromocenters as the synaptonemal complex lengthens. Second, in mutants that are incapable of synapsis, the homologous axes still align, but the centromeres remain in chromocenters with non-homologous partners. However, synapsis cannot be the only mechanism controlling this chromocenter re-organization. After complete synapsis in pachytene, in the transition to diplotene, the chromocenters continue to individualize, moving from clumps of homologous centromere pairs to mostly single homologous centromere pairs. It is not known what drives this resolution of the centromere clusters to individual pairs. But the pericentric heterochromatin in the chromocenters is rich in cohesin, condensin, and topoisomerase II (Ishiguro et al. 2011; Verver et al. 2013; Gómez et al. 2013; Ishiguro et al. 2014; Hopkins et al. 2014). It may be that the interplay of these chromatin compaction factors is important for regulating the formation and dissolution of chromocenters. Consistent with this notion, mutation of the cohesin gene *Stag3* in mice increases the number of chromocenters suggesting that cohesins are necessary for holding together the pericentric heterochromatin of multiple chromosomes (Hopkins et al. 2014).

### Origin and regulation of homologous heterochromatin connections

When and how are homologous heterochromatin connections established? Our results define a period of prophase I in which the SC promotes the stable homologous pericentromeric heterochromatin interactions observed between diplotene chromosomes. First, prior to synapsis, spermatocytes display high numbers of non-homologous centromeres connected by heterochromatin. Second, diplotene *Sycp1*^*−/−*^ spermatocytes have abnormally high numbers of unpaired chromosomes and chromosomes engaged in non-homologous centromeric associations, suggesting the SC plays a role in establishment of homologous heterochromatin connections. However, once homologous centromeres have been juxtaposed by synapsis, the SC is no longer necessary to maintain the association of the homologous pericentromeric heterochromatin regions, since precocious removal of the SC from paired centromeres of diplotene chromosomes (using a PP2A inhibitor) disrupted the close juxtaposition of homologous centromeres (centromere pairing) but did not affect heterochromatin interactions between homologous pairs. Thus, there must be a mechanism that stabilizes heterochromatin connections between homolog pairs independently of the SC.

What is the nature of heterochromatin interactions and what activity could disrupt heterochromatin connections from homologous centromeres when they segregate? The tight physical association of heterochromatin observed in *Drosophila* oocytes during early meiosis suggested the possibility that heterochromatin connections may be established during DNA replication (Dernburg et al. 1996). It has been suggested that linkages are established during stalled replication fork repair (Hughes et al. 2009); however, our results suggest that the coalescence of pericentric heterochromatin into chromocenters containing multiple centromeres does not happen until well after S-phase.

The persistence of heterochromatic associations into meiotic pro-metaphase is reminiscent of the ultra-fine DNA threads that connect sister chromatids in mitotic cells (Chan et al. 2009). The connections between mitotic sister chromatid DNAs that are responsible for these threads occur through multiple mechanisms including catenation, late replication intermediates, and telomere fusion events (Liu et al. 2014).

It is possible that protein–protein or protein-DNA interactions of a different nature may promote post-pachytene stable homologous heterochromatin interactions. The pericentromeric heterochromatin of achiasmate partner chromosomes in *Drosophila* is necessary to promote their disjunction in meiosis I. Recent work has suggested that the pericentromeric heterochromatin of homologous chromosomes might become tethered by chromatin proteins (HP1a and Piwi) that recognize heterochromatin methylation marks (Giauque and Bickel 2016). Alternatively, centromeric regions are enriched for cohesion proteins and the roles of different types of meiotic cohesion complexes remain unclear. It is possible that cohesins act to form interhomolog cohesion that links centromeric heterochromatin or, alternatively, provide an environment in which catenation or other links between partner chromosomes are maintained until metaphase. Such a mechanism for linking homologous heterochromatic regions would require a novel meiotic remodeling of cohesins as cells move through prophase. This would include dissolving cohesive links between non-homologous heterochromatin blocks and establishing cohesion between homologous heterochromatin blocks after they are brought together by SC formation. Further work will be necessary to test these hypotheses.

### Experimental procedures

#### Mouse strains

The Oklahoma Medical Research Foundation Animal Care and Use Committee (IACUC) approved all animal protocols. Wild-type (C57BL/6) and *Sycp1*^*−/−*^ mice (de Vries et al., 2005) were used in this study.

#### Cytology

We employed established experimental approaches for the visualization of chromosomes in chromosome surface spreads (Peters et al. 1997). Incubations with primary antibodies were carried out for 12 h at 4 °C in 1× PBS plus BSA 2%. To detect SYCP1 and SYCP3, we used polyclonal antibodies raised against mouse SYCP1 at 1∶150 dilution (Novus Biologicals, NB300-229) and polyclonal chicken antibody generated in our laboratory raised against mouse SYCP3 at 1∶300 dilution. Centromeres were detected using the human centromere protein antibody (CREST, Antibody Incorporated, 9101-02) at 1∶50 dilution. H3K9me3 was detected using polyclonal rabbit antibody raised against H3K9me3 at 1∶500 dilution. Following three washes in 1× PBS, slides were incubated for 1 h at room temperature with secondary antibodies. A combination of fluorescein isothiocyanate (FITC)-conjugated goat anti-rabbit IgG (Jackson laboratories) with Rhodamine-conjugated goat anti-mouse IgG and Cy5-conjugated goat anti-human IgG each diluted 1∶350 was used for simultaneous immunolabeling if required. Slides were subsequently counterstained for 3 min with 2 μg/ml DAPI containing Vectashield mounting solution (Vector Laboratories) and sealed with nail varnish. We used an Axiovision SE 64 (Carl Zeiss, Inc.) for imaging acquisition and processing.

Spermatocyte chromosome spreads for electron microscopy analysis were performed as previously described (Dresser et al. 1987).

For scoring chromosome and centromere behavior, only those chromosomes that could be unambiguously evaluated were included in the analysis. Chromosomes with tangles or covered by other chromosomes were not included in the analyses.

#### Spermatocyte culturing and chemical inhibition

Short-term culture of spermatocytes was performed essentially as described (La Salle et al. 2009). Cantharidin was added at 30 μM (Millipore; 505,156; 30 mM stock dissolved in DMSO) and incubated for 3 h. Cells were then pelleted, washed with 1× PBS and processed for surface spreads. Equivalent volumes of DMSO were added to “no treatment” control cultures.

#### FISH combined with immunostaining

DNA FISH was carried out essentially as previously described (Turner et al. 2005). Cell suspensions were prepared in 1× PBS containing a cocktail of protease inhibitors. Cells were spun down and resuspended in 100 mM sucrose, pH 7.2. Approximately 70 μl of this cell suspension was dropped on clean slides and allowed to attach to the slides for 10 min at RT. Slides were immersed in 4% paraformaldehyde for 20 min and rinsed in 1× PBS, dehydrated through an ethanol series (2× 70%, 80%, 96%, 100%), and air-dried. Hybridization solution-specific fluorescent point probes for chromosomes 2, 8, and 15 were obtained from ID Labs Inc. Samples were incubated in humid chambers for 24 h at 37 °C. We then subjected slides to washes at 42 °C (three washes with 2× SSC and 50% formamide and three washes with 2× SSC) and transferred them to 4× SSC and 0.1% Tween-20. Slides were blocked in 4× SSC, 4 mg/ml bovine serum albumin, and 0.001% Tween-20 for 30 min at 37 °C. At each of these steps, the slides were incubated for 30 min at 37 °C and washed three times for 2 min each in 4× SSC and 0.1% Tween-20. Slides were cross-linked with 1% PFA/1× PBS for 10 min and immunostained with the corresponding antibody.

#### Statistical tests

The statistical tests are described in the text and figure legends. Statistical tests were performed using Prizm software.

## Electronic supplementary material

ESM 1 (DOCX 181 kb)
