# Supplementary material for: The pericentromeric heterochromatin of homologous chromosomes remains associated after centromere pairing dissolves in mouse spermatocyte meiosis
Source: Chromosoma. 2019 Jun 4;128(3):355–67. doi: 10.1007/s00412-019-00708-6 (PMC6823320; doi:10.1007/s00412-019-00708-6)
Supplement: Supplementary file 1 — (DOCX 181 kb) [file 412_2019_708_MOESM1_ESM.docx]

**Supplemental Material For:**

**Heterochromatin Interactions Maintain Homologous Centromere Associations in Mouse Spermatocyte Meiosis**

Hoa H. Chuong^1^, Craig Eyster^1^, Chih-Ying Lee^1^, Roberto J. Pezza^1, 2, 3^, and Dean Dawson^1, 2, 3^

^1^Oklahoma Medical Research Foundation, Oklahoma City, Oklahoma, United States of America.

^2^Department of Cell Biology, University of Oklahoma Health Science Center, Oklahoma City, Oklahoma, United States of America.

^3^co-corresponding authors

**Figure S1. Pattern of pericentromeric organization through meiotic prophase.** Squash preparations like those shown in Figure 1 A were scored to determine the timing of pericentromeric chromatin re-organization events in early meiosis. Cells were staged according to their nuclear and chromosomal morphologies. **A.** The numbers of chromocenters per nucleus were scored. Chromocenters are large and in small numbers in leptotene in significantly decrease in number as cells progress. **B.** The number of CREST staining foci per nucleus. As synapsis proceeds (leptotene to pachytene) the numbers of CREST foci drop significantly consistent with homologous centromere pairs being brought into close juxtaposition such that the pair yields a single CREST focus. **C.** The number of chromocenters was scored for pachytene-like (aligned SYCP3 axes) *Sycp1^-/-^* cells. For the above graphs, the number of nuclei scored for each timepoint are: Spermatogonia B, 53: S-phase, 72: Leptotene, 23: Zygotene, 70; Pachytene, 36; Diplotene, 47; *Sycp1^-/-^*, 11. Upaired t-tests were used for statistical comparisons. ****p<0.0001.
